# Diagnosing acute and prevalent HIV-1 infection in young African adults seeking care for fever: a systematic review and audit of current practice

**DOI:** 10.1093/inthealth/ihu024

**Published:** 2014-05-19

**Authors:** Henrieke A.B. Prins, Peter Mugo, Elizabeth Wahome, Grace Mwashigadi, Alexander Thiong'o, Adrian Smith, Eduard J. Sanders, Susan M. Graham

**Affiliations:** a Kenya Medical Research Institute, Centre for Geographic Medicine Research–Coast, P.O. Box 230, Kilifi, Kenya; b Nuffield Department of Population Health, University of Oxford, Oxford OX3 7BN, UK; c Nuffield Department of Clinical Medicine, University of Oxford, Oxford OX3 7BN, UK; d University of Washington, Seattle, WA 98195, USA

**Keywords:** Acute disease, Adults, Fever, HIV-1, Outpatients, Sub-Saharan Africa

## Abstract

Fever is a common complaint in HIV-1 infected adults and may be a presenting sign of acute HIV-1 infection (AHI). We investigated the extent to which HIV-1 infection was considered in the diagnostic evaluation of febrile adults in sub-Saharan Africa (SSA) through a systematic review of published literature and guidelines in the period 2003–2014. We also performed a detailed audit of current practice for the evaluation of febrile young adults in coastal Kenya. Our review identified 43 studies investigating the aetiology of fever in adult outpatients in SSA. While the guidelines identified recommend testing for HIV-1 infection, none mentioned AHI. In our audit of current practice at nine health facilities, only 189 out of 1173 (16.1%) patients, aged 18–29 years, were tested for HIV-1. In a detailed record review, only 2 out of 39 (5.1%) young adults seeking care for fever were tested for HIV-1, and the possibility of AHI was not mentioned. Available literature on adult outpatients presenting with fever is heavily focused on diagnosing malaria and guidelines are poorly defined in terms of evaluating aetiologies other than malaria. Current practice in coastal Kenya shows poor uptake of provider-initiated HIV-1 testing and AHI is not currently considered in the differential diagnosis.

## Introduction

Routine HIV-1 screening of adults in healthcare settings provides an effective strategy to identify and potentially present into care large numbers of previously undiagnosed HIV-1-positive individuals.^1,2^ The importance of such screening is heightened in the current era of widespread antiretroviral therapy (ART) availability. To ensure that screening is carried out, the Joint United Nations Programme on HIV-1/AIDS issued guidance in 2007 on provider initiated testing and counselling (PITC).^3^ Since then, PITC has been recommended as a routine part of medical care in areas with generalised HIV-1 epidemics. For example, Kenya's National AIDS and STI Control Programme adopted PITC in 2008, and issued National Guidelines for HIV-1 counselling and testing at government health facilities.^4^

In 2013, the 2012 Kenya AIDS indicator survey reported a steady increase in HIV-1 prevalence with age, from 2.1% among men and women aged 15–24 years to 6.4% among those aged 25–34 years, peaking at 9% among those aged 35–54 years.^5^ Unfortunately, despite the steady increase in HIV-1 prevalence with age, data from routine programmes in sub-Saharan Africa (SSA) show that the proportion of patients offered an HIV-1 test has been disappointingly low.^6^ Furthermore, PITC by design screens for prevalent HIV-1 infection using low-cost point-of-care (POC) rapid tests. As such, PITC fails to identify patients with an acute HIV-1 infection (AHI) who seek healthcare. This is of public health concern as patients with AHI, during the first 3–4 weeks following HIV-1 acquisition, are highly contagious.^7,8^ Low uptake of PITC and failure to consider the diagnosis of AHI in febrile patients both seriously reduce the impact of current provider-initiated testing programmes.

To assess current knowledge and practice of HIV-1 and AHI testing among febrile adults in SSA, and in particular in Kenya, we carried out a study in two parts: the first focusing on evidence and guidelines and the second focusing on practice. In part one, we performed a systematic review of literature describing diagnostic testing of adults presenting with fever at outpatient clinics in SSA. We also assessed whether currently available guidelines and recommendations reflect the most recent literature. In part two, we set out to investigate current practice regarding the management of young adults presenting with fever at health facilities in coastal Kenya, with a particular focus on whether evaluation included testing for acute or prevalent HIV-1 infection.

## Methods

### Systematic review

In consultation with the Bodleian Library of Oxford, a systematic review of evidence-based literature published between 1 January 2003 and 16 February 2014 was performed. Publications reporting the evaluation of adults seeking healthcare for fever at outpatient clinics in SSA were identified through online searches of PubMed, Embase, Web of Science, Global Health, the Cochrane Library, JSTOR, ScienceDirect, Google Scholar and Africa Index Medicus. Databases were searched using the keywords ‘fever’ and ‘febrile’ combined with the targeted age group (adults), clinical setting (outpatient) and the geographic area (SSA) in all fields. The search was limited to papers published in the past decade, under the assumption that evidence published before this period was less pertinent to current guidelines. Searching all databases did not result in the identification of additional publications beyond what was found in PubMed, Embase and Web of Science. Therefore, the latter three databases were used. For an overview of the search strategy and an example of one full electronic search (PubMed), see Supplementary data.

**Figure 1.**
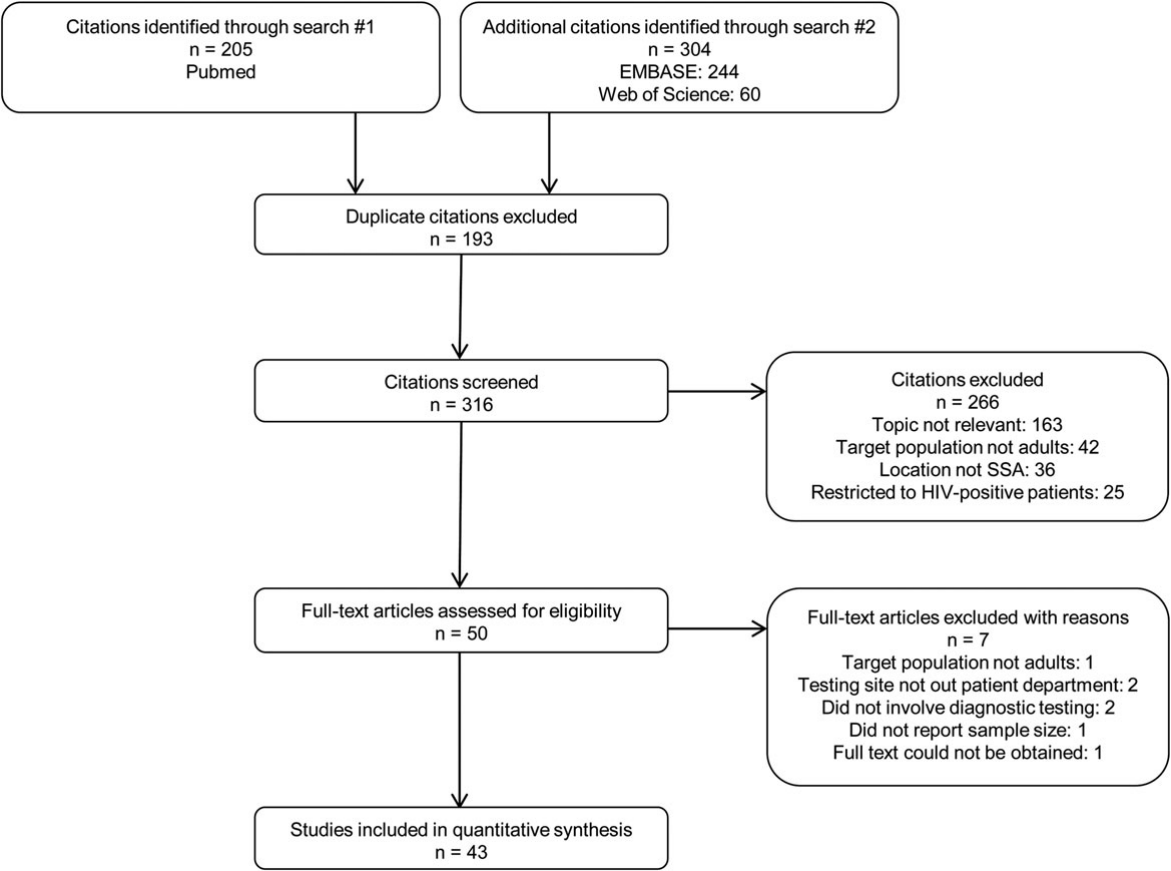
Results of the search and screening process. Template adapted from PRISMA 2009 flow diagram. SSA: sub-Sahara Africa.

All citations resulting from this search were included for screening. The search results from each database were collated and duplicates were removed. Articles were excluded if they: 1. did not address outpatients presenting with fever; 2. were restricted to HIV-1-positive patients, pregnant women, neonates or children; or 3. were conducted outside SSA. Two independent reviewers (PM and HABP) evaluated each citation for inclusion. First, titles and abstracts were screened. Full-texts were retrieved for articles that looked eligible on screening of the title and abstract and for records that did not contain sufficient information in the abstract for relevance to be accurately judged. Figure 1 summarises the search and screening process. The final lists of citations for inclusion were compared and any discrepancies between them were resolved by discussion and consensus of all authors.

For each included study, a standard data-collection form was used to record the setting, population, study design, methods, results and whether testing for acute or prevalent HIV-1 infection was performed. From the published data, we derived the screening prevalence of the aetiologies that were tested for in each study. Where information on methods or specific results relevant to the systematic review was not described in the published manuscripts, authors were contacted for details. When possible, for studies including both adults and children, we disaggregated data for adults only. In addition, we obtained data on HIV-1 prevalence in each study location (at the country level) using currently published figures from the WHO.^9^

### Guideline review

Guidelines or recommendations regarding management of febrile young adults were identified through searching the online public access WHO guidelines database^10^ and the Turning Research Into Practice (TRIP) online database.^11^ The WHO database was searched using the keywords ‘fever’ and ‘febrile illness’, ‘management’ and ‘outpatient’, including ‘adult’ and excluding ‘children’ in the WHO regional site of Africa. The TRIP database was searched using the same strategy as that used in PubMed, Embase and Web of Science. For an overview of the search strategy and an example of one full electronic search (TRIP) see Supplementary data. In addition, we obtained curricula used for training clinical officers and nurses by the Kenya Medical Training Colleges. The retrieved recommendations and guidelines were screened independently by PM and HABP for sections on management of fever. Discrepancies were resolved through discussion with all clinical authors.

### Audit of current practice

In part two of this study, we first conducted an audit of current HIV-1 testing practice for adults <30 years of age, as this young group has the highest HIV-1 acquisition risk.^12^ The audit was carried out in Mtwapa and Shanzu, two adjacent coastal towns near Mombasa. The estimated population of these two towns is approximately 100 000, and 26 healthcare facilities, including 4 government facilities, have been identified in 2011.^13^ From this existing list of care providers, we approached all four government facilities and randomly selected six private facilities to participate in the audit, using computer-generated random number selection. The clinician in-charge was requested to participate in the audit and gave consent using an informed consent document. One government facility refused to participate because official permission could not be obtained, and all private facilities accepted. Informed consent documents and the protocol for the audit of current practice were approved by the Kenya Medical Research Institute scientific and ethical review committees.

Capacity to test for acute and prevalent HIV-1 infection, and the use of any guidelines for management of febrile illness or any algorithm for AHI diagnosis, were both assessed. Uptake of PITC for young adults was determined by the total number of HIV-1 tests conducted among adult patients aged <30 years divided by the total number of adult patients within this age group who attended the facility in the corresponding period. Records kept at government clinics did not allow for in-depth review, because individual patient encounters were not detailed. At each of the six private health facilities, medical records of up to 12 consecutive patients seeking healthcare for fever were reviewed by facility clinicians using an anonymised data extraction form. After data collection, up to three representatives from each facility were invited for a focus group discussion (FGD) to discuss the management of adult patients presenting with fever and the uptake of PITC. All data collected were analysed using STATA version 11 software (Stata Corporation, TX, USA).

## Results

### Literature review

Figure 1 depicts results from the literature search and screening process. After removal of 193 duplicates, 316 citations were screened independently. A total of 266 citations were removed due to irrelevance to the study topic or other exclusion criteria. Subsequently, 50 full-text articles were assessed for eligibility. Of these, seven articles were excluded after detailed review: two studies that did not involve testing at outpatient departments, two studies that did not include diagnostic testing, one study that included only patients under 17 years of age, one study that presented only percentages without a study population size and one study for which the full text could not be obtained.

Table 1 presents a detailed overview of the data extracted from the 43 full texts included in the final analysis. The identified studies investigated the following fever aetiologies: malaria only (34 studies), acute HIV-1 infection and malaria (2 studies), human herpes virus-8 and hepatitis B virus (1 study), pneumococcal bacteraemia (1 study), flaviviruses (1 study), influenza viruses (1 study), Lassa virus (1 study), intestinal helminths (1 study) and schistosomiasis (one study). The most common study design was cross-sectional (31/43), and the majority of studies were carried out in East Africa (27/43). Only two studies with primary interest in acute HIV-1 infection tested all study participants for HIV-1. One additional study performed limited HIV-1 testing (2 of 190 febrile patients aged 5 years and above were assessed). In 13 studies, HIV-1 testing was not performed at all. Information on HIV-1 testing could not be obtained for 27 studies.

**Table 1.** Studies of fever aetiology in adults presenting with febrile illness in sub-Saharan Africa

Author (reference)	Year	Country	Setting	Study design	Study population: total, adults (N, n)^a^	Participant age^b^	Definition of fever	Estimated HIV-1 prevalence in study area^c^ (CI)	Study HIV-1 test results	Study malaria test results^d^	Other causes investigated and study test results
Arness^28^	2003	Kenya	P	CS	2796	0.3–85^b^	Temperature of >38.2°C or clinical signs suggestive of malaria	7.6 (7.3–7.9)	NS	28.5% (798/2796)	None
Wang^29^	2005	Burkina Faso	FB, P, G	CS	6109, 266	>15	Temperature ≥37.5°C or history of fever in the past 36 h	1.4% (1.2%–1.6%)	NS	18.0% (48/266)	None
Wang^30^	2006	Tanzania	G, P	CS	1498, 308	>15	Temperature ≥37.5°C or history of fever in the past 36 h	6.0% (5.4%–6.6%)	NS	4.2% (13/308)	None
Wang^31^	2006	Côte d'Ivoire	G, P	CS	812, 120	>15	Temperature ≥37.5°C or history of fever in the past 36 h	4.6% (4.1%–5.2%)	NS	25.6% (31/120)	None
Wang^32^	2006	Benin	G, P	CS	1263, 213	>15	Temperature ≥37.5°C or history of fever in the past 36 h	1.3% (1.2%–1.5%)	NS	0.9% (2/213)	None
Reyburn^19^	2006	Tanzania	G	CS	1273, 214	All ages^b^	Suspected malaria	6.0% (5.4%–6.6%)	ND	6.1% (13/214)	None
Zurovac^22^	2006	Kenya	G	CS	359, 222	≥5^a^	Axillary temperature of ≥37.5°C or a history of fever in the last 48 h	6.6 (6.3–6.8)	ND	44.6% (99/222)	None
Reyburn^33^	2007	Tanzania	G	RT	2416, 1043	>15	Suspected malaria	5.8% (5.2%–6.3%)	ND	8.1% (84/1043)	None
Ndyomugyenyi^34^	2007	Uganda	G	CS	1627, 1165	≥16	Axillary temperature of ≥37.5°C or history of fever in the last 24 h	6.7% (5.9%–7.7%)	ND	24.8% (289/1165)	None
Rakotonirina^35^	2008	Madagascar	G	CS	313, 95	>18	Axillary temperature of ≥37.5°C or history of fever in the last 24 h	0.6% (0.5%–0.7%)	ND	16.8% (16/95)	None
Pfeiffer^36^	2008	Burkina Faso	G	CS	1101, 1019	0.08–86^b^	Axillary temperature of ≥37.5°C or history of fever	1.1% (0.9%–1.3%)	ND	70.5% (718/1019)	None
Chandler^37^	2008	Tanzania	FB, G	PO	2082, 13	>15	Clinical features suggestive of malaria: current or recent history of fever	5.7% (5.1%–6.2%)	ND	30.8% (4/13)	None
Kyabayinze^38^	2008	Uganda	G	CS	357, 194	>5^b^	Axillary temperature ≥ 37.5°C or a history of fever in the last 24 h or; no evidence of a concomitant febrile illness; no danger signs or evidence of severe malaria	6.7% (5.9%–7.7%)	NS	19.1% (37/194)^BS^, 51.5% (100/194)^RDT^	None
Mwanziva^21^	2008	Tanzania	FB	PO	240, 176	≥18	Symptoms suggestive of malaria	5.7% (5.1%–6.2%)	ND	36.4% (64/176)^BS^, 4.0% (7/176)^RDT^	None
Nicastri^20^	2009	Tanzania	G	CS	336, 170	≥15	Temperature of >38°C for <10 days	5.7% (5.1%–6.2%)	NS	10.0% (17/170)^BS^, 5.3% (9/170)^RDT^	None
A-Elgayoum^39^	2009	Central Sudan	G	PR	410	All ages^b^	Suspected malaria	NA	NS	6.8% (28/410)	None
Ampofo^40^	2009	Ghana	G	PR	768	All ages^b^	Temperature of >38°C and at least cough, sore throat,coryza, myalgia and headache	1.6% (1.4%–1.8%)	NS	NS	Influenza: 7.4% (57/768)
Bisoffi^41^	2009	Burkina Faso	G	CC	5236, 2169	≥0.5^b^	Temperature of ≥37.5°C	1.1% (0.9%–1.2%)	NS	47.3% (1027/2169)	None
Sayang^42^	2009	Cameroon	FB	PR, CC	313	≥11^b^	Axillary temperature of ≥37.5°C or history of fever in the last 24 h	4.7% (4.4%–5.1%)	NS	36.1% (113/313)	None
Rowe^17^	2009	Angola	G	CS	177, 39	≥18	Suspected malaria defined as axillary temperature of ≥37.5°C	2.2% (1.8%–2.7%)	ND	0% (0/39)	None
Thwing^43^	2009	Angola	G	CS	864, 250	≥15	Axillary temperature ≥37.5°C or history of fever in the past 24 h, without signs of severe illness	2.2% (1.8%–2.7%)	NS	4.0% (10/250)	None
Meschi^44^	2010	Tanzania	G	CS	366, 137, 133	>18	Temperature of >38°C for <10 days	5.5% (5.0%–6.1%)	ND	ND	Acute HHV-8: 1.5% (2/137), acute HBV: 2.3% (3/133)
Bebell^14^	2010	Uganda	G	CS	2893, 2888	≥13^b^	Patients referred for malaria BS	7.0% (6.3%–8.2%)	HIV-1: 8% (238/2888); EHI: 1.9% (56/2888); AHI: 1.0% (30/2888)	17.1% (494/2888)	None
Chinkhumba^45^	2010	Malawi	G	CS	2576, 2573	≥5^b^	Documented fever or a history of fever in the previous 24 h	11.2% (10.6%–11.8%)	NS	25% (643/2573)^BS^, 56% (1441/2573)^RDT^	None
Serna-Bolea^15^	2010	Mozambique	G	PC	472, 346	18–86^b^	Self reported fever	11.4% (10.2%–13.0%)	HIV-1: 38% (131/346); AHI: 3.3% (7/211)	16.2% (56/346)	None
Feikin^46^	2010	Kenya	FB	PC	21000, 190	≥5^b^	Temperature of ≥38°C	6.2 (6.0–6.4)	HIV-1: 0% (0/2), targeted testing only	ND	Bacteraemia: 2.1% (4/190); pneumococcus: 1.1% (2/190); HIV-1: 0% (0/2)
Okebe^47^	2010	The Gambia	G	CS	521, 168	>15	Suspected or confirmed malaria	1.3% (1.0%–1.7%)	NS	7.7% (13/168)	None
Juma^48^	2011	Kenya	FB, G, NGO	CS	1096, 284	≥5^b^	Axillary temperature of ≥37.5°C or a history of fever during the present illness	6.2 (5.9–6.3)	ND	58.1% (165/284)	None
Kahama-Maro^49^	2011	Tanzania	G	CS	346, 265	>15	Axillary temperature of ≥37.5°C or history of fever in the preceding 48 h	5.3% (4.7%–5.8%)	NS	14.3% (38/265)	None
Macedo de Oliveira^50^	2011	Mozambique	G	CS	706	0.3–84^b^	Axillary temperature of ≥37.5°C or history of fever in the last 24 h	11.2% (10.0%–13.0%)	NS	15.7% (111/706)	None
Oduro^51^	2011	The Gambia	NS	CS	16230, 2191	All ages^b^	Temperature of ≥37.5°C	1.3% (1.0%–1.7%)	NS	17.2% (376/2192)	None
Ouldabdallahi^16^	2011	Mauritania	G	CS	1431, 427	>15	Suspected malaria	0.5% (0.4%–0.7%)	NS	6.6% (28/427)	None
Batwala^52^	2011	Uganda	G	Cluster RT	52116, 9313	≥5^b^	Axillary temperature of ≥37.5°C or history of fever	7.2% (6.4%–8.3%)	ND	13.7% (1279/9313)^BS^; 20.0% (2730/13624)^RDT^	None
Vairo^53^	2012	Tanzania	G	CS	202	≥15	Temperature of >38°C for <10 days	5.1 (4.6–5.7)	ND	ND	No CHIKV, no acute DENV
Bevilacqua^54^	2012	Tanzania	G	CS	297, 177	≥1^b^	Temperature of >38°C for <10 days	5.1 (4.6–5.7)	NS	NS	Schistosomiasis: 12.4% (22/177)
Ehichioya^55^	2012	Nigeria	G	CS	451	All ages^b^	Fever	3.1% (2.8%–3.5%)	NS	NS	Lassa Fever: 0.4% (2/451)
Mangham^56^	2012	Cameroon	P, PU	CS	2039, 456	All ages^b^	Seeking treatment for a fever or having received an artemisinin combination therapy	4.5% (4.1%–4.9%)	NS	32.7% (149/456)	None
Sleshi^57^	2012	Ethiopia	FB, G, P,	CS	260	1–62^b^	Symptoms of uncomplicated malaria, temperature ≥37.5°C or history of fever in the past 48 h	1.3% (1.2%–1.5%)	NS	19.6% (51/260)	ND
Baltzell^58^	2013	Tanzania	G	PR	594, 228	>18	Axillary temperature of ≥37.5°C or history of fever in the preceding 24 h, and absence of any danger signs of severe disease	NA	NS	3.5% (8/228)	None
Bruxvoort^59^	2013	Tanzania	G	CS	3456	All ages^b^	Fever or history of fever in the previous 48 h	NA	NS	12.8% (443/3456)^BS^, 21.8% (755/3456)^RDT^	None
Mubi^60^	2013	Tanzania	G	CS	168, 48	>5^b^	Fever or history of fever	NA	NS	29.2% (14/48)	None
Njozi^18^	2013	Tanzania	G	Observational, longitudinal and prospective	11648, 5076	All ages^b^	Fever or history of fever and treated with artemether-lumefantrine	NA	NS	79.1% (4013/5076)	None
Degarege^61^	2014	Ethiopia	G	CS	1065, 570	>15	Acute febrile patients suspected of malaria	NA	NS	20.4% (116/570)	Intestinal helminth infection: 52.8% (228/432)

AHI: acute HIV-1 infection; CC: case control; CHIKV: chikungunya virus; CS: cross-sectional; DENV: dengue virus; EHI: early HIV-1 infection; FB: faith-based; G: government; HBV: hepatitis B virus infection; HHV-8: human herpesvirus-8; NA: not available; ND: not done; NGO: non-governmental organisation; NS: not specified; P: private; PC: prospective cohort; PO: prospective observational; PR: prospective; PU: public; RT: randomised trial.

^a^ Study population defined as the number of outpatients analysed (N), number of febrile children or adults tested (n) (if different from N).

^b^ Studies where data specific to patients aged 18 years or older could not be retrieved from either the published article or from the authors in personal communication.

^c^ Estimated HIV-1 prevalence per country in the year of publication as indicated by UNAIDS AIDS Info.^9^

^d^ The screening prevalence is based on peripheral blood slides or rapid diagnostic test. If more than one diagnostic test was used, the test is indicated. Where both fieldworkers or clinicians and expert researchers performed diagnostic tests, the screening prevalence was calculated using test results from fieldworkers or clinicians.

Both studies that included testing for acute and prevalent HIV-1 also tested participants for malaria.^14,15^ In the first of these studies, Bebell et al. investigated acute, early and prevalent HIV-1 infection among patients aged 13 years or older with suspected malaria in an outpatient setting in rural Uganda.^14^ To identify acute and early HIV-1 infection the following diagnostic tests were performed on dried blood spots: HIV-1 RNA nucleic acid amplification, HIV-1 antibody immunoassay, HIV-1 antibody western blot and enzyme immunoassay (EIA). Acute HIV-1 infection was defined as detectable HIV-1 RNA with a negative or indeterminate HIV-1 western blot pattern. Early HIV-1 infection was defined as detectable HIV-1 RNA with a positive western blot pattern and a detuned EIA result (Calypte HIV-1 BED Incidence EIA, Calypte Biomedical, Portland, OR, USA) compatible with early infection. Of 2893 outpatients aged 13 years and above who were referred for a malaria blood slide, 238 (8.2%; 238/2893) were diagnosed with prevalent HIV-1 infection, and 56 (1.9%; 56/2893) were diagnosed with early HIV-1 infection. Of the 2599 HIV-1 seronegative patients, 30 (1.2%; 30/2599) were found to have AHI. Among the 2893 patients evaluated, 494 (17.1%; 494/2893) had blood smears positive for malaria parasites. Two (0.4%; 2/4893) of the AHI cases were also malaria positive. In the second study, Serna-Bolea et al. determined the prevalence of AHI among adult patients (all 18 years of age or older) presenting with fever in a district hospital outpatient ward in southern Mozambique.^15^ After rapid tests were used to identify HIV-1-seropositive patients, pooled plasma HIV-1 RNA testing was conducted for all HIV-1 seronegative patients. Acute HIV-1 infection was defined as detectable HIV-1 RNA in a seronegative patient. Of 346 patients aged 18 years and above, 135 (39.0%; 135/346) were diagnosed with established HIV-1 infection. Of the 211 HIV-1 seronegative patients, 7 (3.3%; 7/211) were found to have AHI. Among the 346 adults evaluated, 56 (16.2%; 56/346) had a positive *Plasmodium falciparum* rapid test. None of the AHI cases were also malaria positive.

Table 1 shows that national HIV-1 prevalence estimates ranged from 0.5% in Mauritania in 2011 to 11.4% in Mozambique in 2010.^15,16^ The screening prevalence for malaria in the 37 studies that tested for this aetiology ranged from 0% in Angola in 2009 to 79.1% in Tanzania in 2013.^17,18^ Of note, in those studies where both health workers and expert researchers performed diagnostic tests, or test results were PCR confirmed, positivity rates for blood slides judged by health workers were consistently higher than those judged by research experts or PCR rates (data not shown).^19–22^

### Guideline review

A search for guidelines regarding management of fever in the online public-access WHO guidelines database resulted in ‘Acute Care: Integrated Management of Adolescent and Adult Illness (IMAI)’ guidelines for first-level facility health workers at health centres and district outpatient clinics, first published in 2004 and updated in 2009.^23^ The guideline recommends considering ‘HIV-1 related illness if unexplained fever for >30 days' in light of a negative malaria smear or dipstick. In febrile patients in who the diagnosis of malaria has been excluded, the Acute Care IMAI recommends: ‘treat according to apparent cause.’ No national guidelines for the outpatient management of adults presenting with fever were found. No sections on management of non-severe febrile illness in adults not previously known to be HIV-1-positive were found in the Kenya Medical Training Colleges curricula employed.

### Audit of current practice

**Figure 2.**
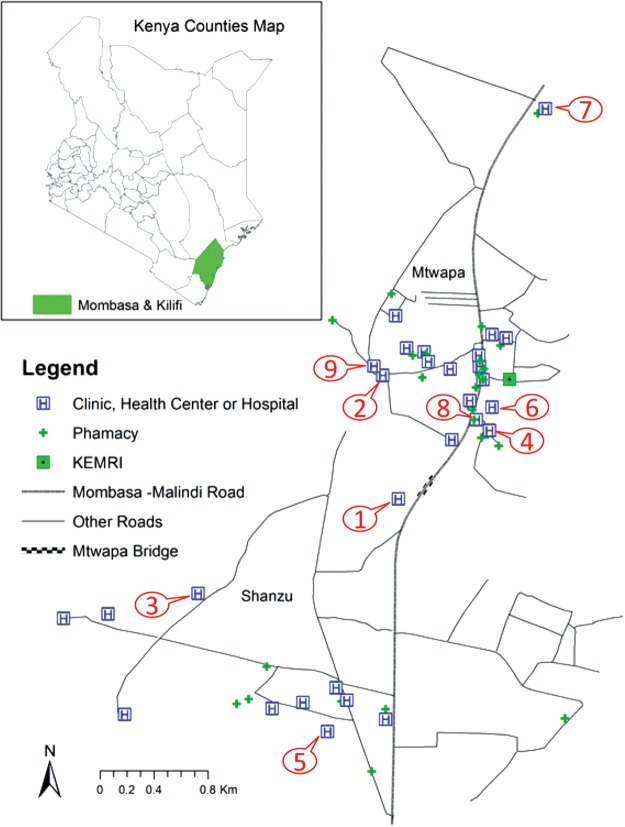
Health facilities included in the audit of current practice. Numbers on the map correspond to numbers as listed in 2.

Table 2 presents the characteristics, AHI diagnostic capacity and PITC rates of the nine health facilities included in the audit of current practice. Figure 2 shows a map of the study area depicting the nine facilities included in the audit. During the audit period (6 to 17 August 2012), 1173 patients who were 18–29 years of age visited nine health facilities in coastal Kenya as outpatients seeking care for a number of complaints. PITC was performed for 189 (16.1%; 189/1173) of these patients. Testing rates were similar at government and private health facilities (15.4% and 18.4%, respectively, p=0.3) (data not shown). Facility records did not record instances where PITC was offered but refused by the patient or not offered due to service constraints. Capacity for HIV RNA, p24 or detuned ELISA testing was not available in any of these facilities, and none had an algorithm for AHI diagnosis.

**Table 2.** Characteristics, acute HIV-1 diagnostic capacity and provider initiated testing and counselling (PITC) rates of the nine health facilities in the audit of current practice

Health facility number	Health facility type	No. patients seen^a^	Patients 18–29 years	Patients 18–29 years tested for HIV, n (%)	HIV prevalence among those tested	Charts audited^b^	AHI in differential diagnosis of febrile patients	AHI diagnostic capacity^c^
1	G	1058	503	38 (7.6%)	5.3%	0^e^	No differential	No
2	G	341	205	89 (43.0%)^d^	3.4%	0^e^	No differential	No
3	G	384	226	18 (8.0%)	11.1%	0^e^	No differential	No
4	P	129	47	7 (14.9%)	0.0%	12	No	No
5	P	30	25	0	—	12	No	No
6	P	68	29	7 (24.0%)	0.0%	10	No	No
7	P	240	121	30 (24.8%)	13.3%	9	No	No
8	P	22	0	0	—	12	No	No
9	P	40	17	0	—	0^e^	No differential	No
Total		2312	1173	189 (16.1%)	5.8%	55	No	No

AHI: acute HIV-1; G: government; P: private; VTC: voluntary counselling and testing.

^a^ Outpatients aged 18–29 years who visited the nine health facilities seeking care for various complaints during 6 to 17 August 2012.

^b^ Charts recording fever were audited for patients visiting the six private health facilities during 4 August 2012 to 22 January 2013.

^c^ AHI diagnostic capacity: antigen or RNA detection method or detuned ELISA.

^d^ HIV-testing at this facility included PITC and VCT (data could not be separated).

^e^ Government facilities and one private facility did not keep detailed records of individual patient encounters.

The audit of consecutive cases of documented fever (4 August 2012 to 22 January 2013) at each of the six private health facilities resulted in a total of 66 patient record reviews. Of these, 39 records met our age criteria of 18–29 years. Only 2 (5.1%; 2/39) were tested for HIV-1. A working diagnosis was reported in 36 records: fever was presumed to be due to malaria in 20 (55.6%; 20/36) cases, while 9 (25.0%; 9/36) patients were suspected to have a common bacterial or viral infection. Other working diagnoses included: tonsillitis (n=4), fatigue (n=2) and gastritis (n=1). Of the 20 febrile patients with a working diagnosis of malaria, 16 (80.0%; 16/20) were tested for malaria and 2 (10.0%; 2/20) were tested for HIV-1. Of the nine febrile patients with a working diagnosis of bacterial or viral infection, all were tested for malaria (all tested negative) and none were tested for HIV-1. Table 2 presents information on the number of record reviews, availability of AHI diagnostic tests and use of an AHI diagnostic algorithm at each facility. No facility had AHI diagnostic capacity or used an AHI diagnostic algorithm.

### Focus group discussions with providers

Seven health providers, representing six of the nine invited facilities, attended the FGD conducted after the audit. To manage adult patients presenting with fever, a few clinicians reported using the WHO guidelines on Integrated Management of Childhood Illness, but none reported using the more appropriate WHO guidelines on Integrated Management of Adolescent and Adult Illness (IMAI). One clinician reported considering recent HIV-1 acquisition in the differential diagnosis of the febrile adult, but had no capacity to confirm this diagnosis. All clinicians were aware that current guidelines recommend use of a confirmatory blood slide or rapid diagnostic test to diagnose malaria. With regard to PITC, all providers surveyed reported having received Ministry of Health training. The main challenge reported for the low uptake of PITC was the discrepancy between human resources and demand: clinicians cited high patient loads, the time constraints of pre- and post-test counselling and fluctuations in test kit supplies. Providers pointed out that in case of shortage of testing supplies, priority is given to Voluntary Counselling and Testing (VCT) centres and community outreaches, rather than to PITC. In addition, they noted challenges in convincing patients who have previously tested negative to retest. Furthermore, waiting time at the clinic, duration of the HIV-1 test itself and fear of a positive result were cited as reasons for refusal. At private health facilities, the cost of taking an HIV-1 test was identified as a reason for declining.

## Discussion

The vast majority of studies included in this literature review focussed solely on malaria as a cause of fever. Of the 43 citations, only three involved testing for prevalent HIV-1 infection. Moreover, only two studies indicated AHI as a possible cause of fever. Strikingly, AHI has been found to be as common as confirmed malaria in young adults seeking care for fever in coastal Kenya,^24^ highlighting the need to consider this entity in areas of high HIV-1 transmission. Although fever is a common complaint in adults with a wide range of potential causes, only four studies tested for aetiologies other than prevalent HIV-1 or malaria. Our literature review, therefore, underlines the importance of broadening the differential diagnosis of the adult outpatient presenting with fever.

In a review on this topic published in 2011, Crump et al. recommended that locally relevant research findings and ongoing surveillance should lead to a process of validation, local adaptation and improvement of guidelines for the management of febrile illness.^25^ However, in line with findings from our literature review, current guidelines for the management of fever in SSA remain heavily structured around the diagnosis of malaria. Furthermore, existing guidelines are poorly defined and inadequately disseminated, as exemplified by health workers treating adult febrile patients according to guidelines for childhood illness. For patients with a fever for less than 7 days in whom malaria has been excluded, or for those patients presenting with simple fever who are not at risk for malaria the WHO IMAI algorithm gives no further specification than to ‘treat according to apparent cause.’ HIV-1 testing is not currently recommended as a routine component of evaluation for fever. Moreover, neither the IMAI nor Kenyan National Guidelines recommend including AHI in the differential diagnosis of febrile adult outpatients. Unsurprisingly, in our clinical audit we found no cases in which recent HIV-1 acquisition was suspected.

With respect to PITC, current guidelines universally recommend HIV-1 testing as a routine part of medical care in SSA. As HIV-1 infected adults are more susceptible to infections due to immunosuppression, PITC is especially important for febrile adult patients unaware of their status. Our clinical audit showed low rates of HIV-1 testing among young adults presenting for outpatient care and among young adults presenting with fever. These findings confirm data from routine programmes in SSA that indicate a disappointingly low uptake of PITC.^6^ Providers may lack skills to convince patients to test, and patients may refuse due to added time, extra cost or fear of a positive result. Logistic issues such as long clinic waiting times, inadequate staffing and supply outages further reduce PITC rates. Prioritising PITC for patients with fever or signs of HIV-1 infection could alleviate these problems and might increase current test rates.

With respect to AHI, even if this diagnosis is suspected by clinicians, testing for acute infection is challenged by the current lack of a low-cost POC test for nucleic acid detection in HIV-1-seronegative patients. Convalescent testing using rapid HIV-1 tests 2–4 weeks after the onset of fever, however, can detect seroconversion, and could thus be a potentially cost-effective screening method to diagnose AHI. In addition, laboratory-based p24 antigen testing is feasible in settings where laboratory capacity exists and can detect up to 90% of AHI cases.^26^ Combining rapid HIV-1 testing, p24 antigen testing and repeat rapid testing to detect seroconversion into a pilot AHI screening programme could prove a feasible and scalable approach for the diagnosis of AHI in young, febrile adults in SSA.

Our study has several limitations. First, while we kept the search broad in order to identify all articles on fever in outpatients, we may have missed some studies in which HIV-1 testing was performed in relevant clinical situations. Second, we were unable to disaggregate data on children from data on adults for 21 studies, although we do note that both children and adults should have been tested for HIV, as PITC applies to all age groups. Third, since our count of HIV-1 tests at one of the three government facilities included tests performed at the VTC our calculated testing rate at government facilities is possibly an overestimate. Fourth, there is selection bias in the nine facilities included in our audit of local practice in coastal Kenya, and the limited number of medical records included cover a relatively brief period. Therefore, we may have missed geographic or seasonal variations in clinical practice in coastal Kenya and may not be able to generalise these findings to other countries in SSA. Despite these limitations, our review does indicate that HIV-1 testing and inclusion of AHI in the differential diagnosis are important considerations that should be integrated into clinical management in SSA.

### Conclusions

The results of this review have important policy implications. While the range of potential causes of fever is wide, HIV-1 infection is of significant public health concern, especially in the era of ART scale-up and expanded coverage. Earlier diagnosis of HIV-1 is needed to optimise the impact of guidelines recommending earlier ART initiation. Even for patients who are not yet eligible for ART by current guidelines, co-trimoxazole prophylaxis can decrease morbidity and slow progression to AIDS.^27^ For these reasons, we recommend that the WHO IMAI and Kenyan guidelines be updated to stress the importance of HIV-1 testing in febrile adults, especially in areas with high levels of ongoing HIV-1 transmission. In addition, clinicians should consider AHI in the differential diagnosis of young, sexually-active adults seeking urgent care for fever.

### Supplementary data

Supplementary data are available at International Health Online (http://inthealth.oxfordjournals.org).

## Supplementary Material

Supplementary Data
