# Supplementary material for: Diagnosing acute and prevalent HIV-1 infection in young African adults seeking care for fever: a systematic review and audit of current practice
Source: Int Health. 2014 May 19;6(2):82–92. doi: 10.1093/inthealth/ihu024 (PMC4049276; doi:10.1093/inthealth/ihu024)
Supplement: Supplementary Data [file supp_6_2_82__index.html]

Diagnosing acute and prevalent HIV-1 infection in young African adults seeking care for fever: a systematic review and audit of current practice — Supplementary Data 

# Diagnosing acute and prevalent HIV-1 infection in young African adults seeking care for fever: a systematic review and audit of current practice

## Supplementary Data

Supplementary Data

**Files in this Data Supplement:**

- Supplementary Data - Docx file
