# Supplementary material for: Diagnosing acute and prevalent HIV-1 infection in young African adults seeking care for fever: a systematic review and audit of current practice
Source: Int Health. 2014 May 19;6(2):82–92. doi: 10.1093/inthealth/ihu024 (PMC4049276; doi:10.1093/inthealth/ihu024)
Supplement: Supplementary Data [file supp_ihu024_ihu024supp.docx]

**Supplementary data**

**Electronic search in Pubmed on 16 February 2014**

Search ((((fever OR febrile)) AND (adult OR adolescent OR adult* OR adolescen*)) AND (outpatient OR outpatient clinic OR outpatient ward OR first-level health facility OR health facilit* OR ambulatory)) AND (algeria OR angola OR benin OR botswana OR ’burkino faso‘ OR ’burkina faso‘ OR burundi OR cameroon OR ’cape verde‘ OR ’central african republic‘ OR chad OR comoros OR ’democratic republic of the congo‘ OR ’republic of the congo‘ OR ’cote d'ivore‘ OR djibouti OR egypt OR equatorial guinea OR eritrea OR ethiopia OR gabon OR gambia OR ghana OR guinea OR ’guinea-bissau‘ OR kenya OR lesotho OR liberia OR libya OR madagascar OR malawi OR mali OR mauritania OR mauritius OR morocco OR mozambique OR namibia OR niger OR nigeria OR rwanda OR ’sao tome and principe‘ OR senegal OR seychelles OR ’sierra leone‘ OR somalia OR ’south africa‘ OR ’south sudan‘ OR sudan OR swaziland OR tanzania OR togo OR tunisia OR uganda OR zambia OR zimbabwe OR africa OR ’sub sahara‘ OR ’sub sahara*"’ OR "low resource setting*’ OR "low resource countr*’ OR ’resource limited countr*’ OR "resource limited setting*’ OR ’low-income countr*’ OR ’LIC‘ OR ’developing countr*’ OR ’low income nation‘ OR ’developing nation*’ OR ’less developed countr*’ OR ’less developed nation*’ OR ’least developed nation*’ OR "under developed countr*’ OR ’underdeveloped countr*’ OR ’under developed nation*’ OR "underdeveloped nation*’ OR ’poor countr*’ OR ’third-world nation*’ OR third-world countr*) Filters: Publication date from 2003/01/01 to 2014/02/16.

205 items found.

**Electronic search in TRIP on 16 February 2014**

#19 (#18) from: 2003 to:2014

#18 (#4 AND #9 AND #16 AND #17)

#17 algeria OR angola OR benin OR botswana OR ’burkino faso‘ OR ’burkina faso‘ OR burundi OR cameroon OR ’cape verde‘ OR "central african republic" OR chad OR comoros OR ’democratic republic of the congo‘ OR ’republic of the congo‘ OR ’cote d'ivore‘ OR djibouti OR egypt OR equatorial guinea OR eritrea OR ethiopia OR gabon OR gambia OR ghana OR guinea OR ’guinea-bissau‘ OR kenya OR lesotho OR liberia OR libya OR madagascar OR malawi OR mali OR mauritania OR mauritius OR morocco OR mozambique OR namibia OR niger OR nigeria OR rwanda OR ’sao tome and principe‘ OR senegal OR seychelles OR ’sierra leone‘ OR somalia OR ’south africa‘ OR ’south sudan OR sudan OR swaziland OR tanzania OR togo OR tunisia OR uganda OR zambia OR zimbabwe OR africa OR sub sahara OR sub sahara* OR low resource setting* OR low resource countr* OR resource limited countr* OR resource limited setting* OR low-income countr* OR LIC OR developing countr* OR low income nation OR developing nation* OR less developed countr* OR less developed nation* OR least developed nation* OR under developed countr* OR "underdeveloped countr* OR under developed nation* OR underdeveloped nation* OR "poor countr* OR third-world nation* OR "third-world countr*.

#16 (#10 OR #11 OR #12 OR #13 OR #14 OR #15)

#15 exp outpatient department/

#14 ’ambulatory’

#13 ’outpatient* clinic*’

#12 ’outpatient* ward*’

#11 ’first-level* health* facility*’

#10 ’health facilit*’

#9 (#5 OR #6 OR #7 OR #8)

#8 adolescen*

#7 adult*

#6 adolescent/

#5 adult/

#4 (#1 OR #2 OR #3)

#3 febrile

#2 exp fever/

#1 fever

264 items found’
